# Elevated plasma interleukin-8 as a risk factor for mortality in children presenting with cerebral malaria

**DOI:** 10.1186/s40249-023-01059-2

**Published:** 2023-02-09

**Authors:** Jade Royo, Bertin Vianou, Manfred Accrombessi, Elisée Kinkpé, Linda Ayédadjou, Ida Dossou-Dagba, Yélé Ladipo, Maroufou Jules Alao, Gwladys I. Bertin, Michel Cot, Farid Boumédiène, Sandrine Houzé, Jean François Faucher, Agnès Aubouy, NeuroCM Group, Dissou Affolabi, Daniel Ajzenberg, Bibiane Biokou, Josselin Brisset, Jean-Eudes Degbelo, Philippe Deloron, Latifou Dramane, Sayeh Jafari-Guemouri, Claire Kamaliddin, Anaïs Labrunie, Thomas Lathiere, Achille Massougbodji, Audrey Mowendabeka, Jade Papin, Bernard Pipy, Pierre-Marie Preux, Marie Raymondeau, Darius Sossou, Brigitte Techer, Laurence Watier

**Affiliations:** 1 UMR152 PHARMADEV, IRD, UPS, Toulouse University, 35 Chemin Des Maraichers, 31400 Toulouse, France; 2 Clinical Research Institute of Benin (IRCB), Abomey Calavi, Benin; 3 Faculty of Infectious and Tropical Diseases, Disease Control Department, London School of Hygiene and Tropical Medicine, London, UK; 4 Paediatric Department, Calavi Hospital, Calavi, Benin; 5 Paediatric Department, Mother and Child University and Hospital Center (CHU-MEL), Cotonou, Benin; 6 UMR261 MERIT, IRD, Paris University, Paris, France; 7 UMR 1094 EpiMaCT, Inserm, Limoges University Hospital, Limoges University, Limoges, France; 8 French Malaria Reference Center, APHP, Bichat Hospital, Paris, France; 9 Parasitology Laboratory, APHP, Bichat-Claude-Bernard Hospital, Paris, France; 10 Infectious Diseases and Tropical Medicine Department, Limoges University Hospital, Limoges, France

**Keywords:** Cerebral malaria, Children, Benin, Cytokine, Plasma, Urine, Immunologic marker, NeuroCM

## Abstract

**Background:**

Cerebral malaria (CM) is a neuropathology which remains one of the deadliest forms of malaria among African children. The kinetics of the pathophysiological mechanisms leading to neuroinflammation and the death or survival of patients during CM are still poorly understood. The increasing production of cytokines, chemokines and other actors of the inflammatory and oxidative response by various local actors in response to neuroinflammation plays a major role during CM, participating in both the amplification of the neuroinflammation phenomenon and its resolution. In this study, we aimed to identify risk factors for CM death among specific variables of inflammatory and oxidative responses to improve our understanding of CM pathogenesis.

**Methods:**

Children presenting with CM (*n* = 70) due to *P. falciparum* infection were included in southern Benin and divided according to the clinical outcome into 50 children who survived and 20 who died. Clinical examination was complemented by fundoscopic examination and extensive blood biochemical analysis associated with molecular diagnosis by multiplex PCR targeting 14 pathogens in the patients’ cerebrospinal fluid to rule out coinfections. Luminex technology and enzyme immunoassay kits were used to measure 17 plasma and 7 urinary biomarker levels, respectively. Data were analysed by univariate analysis using the nonparametric Mann‒Whitney *U* test and Pearson’s Chi2 test. Adjusted and multivariate analyses were conducted separately for plasma and urinary biomarkers to identify CM mortality risk factors.

**Results:**

Univariate analysis revealed higher plasma levels of tumour necrosis factor (TNF), interleukin-1beta (IL-1β), IL-10, IL-8, C-X-C motif chemokine ligand 9 (CXCL9), granzyme B, and angiopoietin-2 and lower urinary levels of prostanglandine E2 metabolite (PGEM) in children who died compared to those who survived CM (Mann–Whitney *U*-test, *P*-values between 0.03 and < 0.0001). The multivariate logistic analysis highlighted elevated plasma levels of IL-8 as the main risk factor for death during CM (adjusted odd ratio = 14.2, *P*-value = 0.002). Values obtained during follow-up at D3 and D30 revealed immune factors associated with disease resolution, including plasma CXCL5, C–C motif chemokine ligand 17 (CCL17), CCL22, and urinary 15-F2t-isoprostane.

**Conclusions:**

The main risk factor of death during CM was thus elevated plasma levels of IL-8 at inclusion. Follow-up of patients until D30 revealed marker profiles of disease aggravation and resolution for markers implicated in neutrophil activation, endothelium activation and damage, inflammatory and oxidative response. These results provide important insight into our understanding of CM pathogenesis and clinical outcome and may have important therapeutic implications.

**Graphical Abstract:**

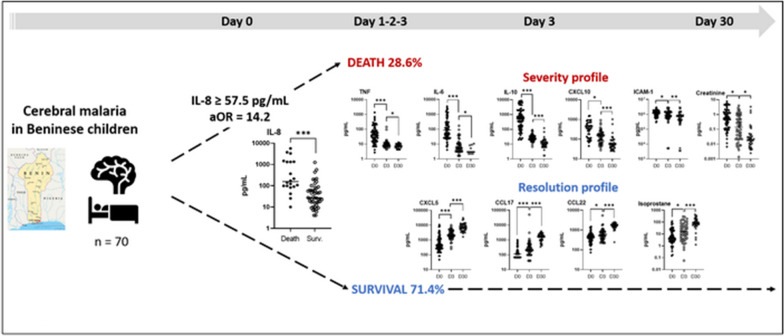

## Background

According to the latest World Health Organization global report, 247 million cases of malaria were reported worldwide in 2021, leading to an estimated 619,000 deaths, 80% of which were in children under 5 years of age in sub-Saharan Africa [1]. Cerebral malaria (CM) is the most severe form of malaria and can lead even when treated to neurological sequelae in 4–10% of cases and death of patients [2]. CM is defined by the presence of the asexual form of *Plasmodium falciparum* parasite associated with a Blantyre score ≤ 2, coma that persists for > 1 h after a seizure, regardless of anticonvulsant medications, and exclusion of any other cause of coma [3]. Today, in endemic countries, the diagnosis of CM is still difficult. A coma in the presence of *P. falciparum* parasites in the peripheral blood is often considered as CM, leading to an overestimation of approximately 25% of CM cases [4]. An accurate differential diagnosis combining fundoscopic examination [5], blood biochemical analysis, and molecular diagnosis of microbial infections would therefore constitute a considerable advance for a diagnosis better adapted to the infectious risks of the area.

The kinetics of the pathophysiological mechanisms leading to the development of CM are still poorly understood. However, infected red blood cells (iRBCs) are known to be sequestered in cerebral blood vessels through cytoadherence to the brain endothelium via parasitic ligands and endothelial receptors such as intercellular adhesion molecule-1 (ICAM-1) and endothelial protein C receptor (EPCR) [6, 7]. This interaction, along with toxin production, leads to the activation of endothelial cells [8]. The latter will then secrete cytokines and chemokines leading to the recruitment of platelets and immune cells to the brain, such as monocytes, lymphocytes and neutrophils [9]. A local inflammatory process is being set [10], with a positive feedback mechanism that leads to the obstruction of cerebral blood flow, increased intracranial pressure, hypoxia and impairment of the integrity of the blood‒brain barrier (BBB).

In this context, the increasing production of cytokines, chemokines and other actors of the inflammatory and oxidative response by various local actors in response to neuroinflammation plays a major role during CM, participating in both the amplification of the neuroinflammation phenomenon and its resolution [9, 11, 12]. Cytokines are membrane or secreted glycoproteins with proinflammatory or anti-inflammatory properties that modulate the local microenvironment. Chemokines are small cytokines capable of activating and recruiting immune cells to the site they are produced and are specifically involved in the recruitment of monocytes/lymphocytes, regulatory lymphocytes and neutrophils [13]. The importance of pro- and anti-inflammatory responses in the pathophysiology of CM has been discussed many times in the literature (reviewed in [14]). If the production of proinflammatory cytokines and chemokines is necessary for the elimination of iRBCs and to achieve an effective immune response, its excessive and uncontrolled development is thought to result in aggravation of neuroinflammation. The resolution of the infection therefore requires a fine adjustment of the kinetics of the production of cytokines, chemokines and other immune markers, although such a strategy still needs to be defined.

The metabolism of arachidonic acid, an unsaturated ω6 fatty acid constituent of the phospholipids of cell membranes, also produces relevant markers of the inflammatory and oxidant responses involved in various non-infectious and infectious diseases [15–18]. Arachidonic acid is metabolized to prostaglandins (PGD2, PGE2, PGF2, PGI2) and thromboxanes by COX1 and COX2 enzymes, to leukotrienes (LTB4, LTC4, LTD4, LTE4) and lipoxins (LXA4, LXB4) by lipoxygenases and to epoxyeicosatrienoic acids via cytochrome p450-catalysed metabolism. Arachidonic acid also produces isoprostanes through nonenzymatic oxidative processes involving free reactive oxygen species (ROS), thereby illustrating a prooxidant environment. Glutathione, involved in leukotrienes and prostaglandins metabolism, is an important actor of the antioxidant response, the reduced glutathione/oxidized glutathione ratio (GSH/GSSG) allowing the evaluation of oxidative stress status. GSH levels were previously shown to be lower in uncomplicated malaria (UM) Ugandan patients compared to healthy controls and decreased in brains from mice presenting experimental CM [19, 20]. Experimental cerebral malaria (ECM) studies have demonstrated the importance of the 5-LOX/LXA4 axis in limiting the proinflammatory and prooxidant response and promoting disease resolution [21, 22]. In humans, it has already been shown that urine PGE2 and COX2 expression are inversely related to disease severity in children, including CM children [23, 24]. Arachidonic acid metabolites are easily measured in urine. We therefore opted for this type of assay, as urine sampling has the advantage of being non-invasive, to provide additional data on these markers of oxidative and inflammatory responses.

We previously demonstrated that predictors of early death in CM are increased bilirubin and lactate, while antibiotic use before admission and vaccination against yellow fever were protective factors [25]. Here, we studied more specific variables of inflammatory and oxidative responses to identify risk factors for CM death to improve our understanding of CM pathogenesis. The added value of our study lies in the quality of the diagnosis used to select subjects with CM that allowed us to rule out nonmalarial comas and coinfections associating *P. falciparum* with a bacterial or viral infection. In addition, for patients who survived, the variables were measured 3 and 30 days (D3, D30) after inclusion in the study. Univariate analysis revealed that higher plasma levels of tumour necrosis factor (TNF), interleukin-1beta (IL-1β), IL-10, IL-8, C-X-C motif chemokine ligand 9 (CXCL9), granzyme B, and angiopoietin-2 and lower urinary levels of PGEM were associated with mortality in CM patients. In a second step, the multivariate logistic regression underlined the strong association between higher plasma IL-8 levels and death outcome during CM. Values obtained during follow-up at D3 and D30 revealed more particularly immune factors associated with disease resolution, including plasma CXCL5, C–C motif chemokine ligand 17 (CCL17), CCL22, and urinary 15-F2t-isoprostane.

## Methods

### Study design and participants

This prospective study was part of the NeuroCM project whose protocol was previously described [26]. Briefly, patients were included from March to December 2018 in two reference hospitals from southern Benin, the Centre Hospitalier Universitaire de la Mère et de l'Enfant Lagune (CHU-MEL) in Cotonou, and the “Centre Hospitalier Universitaire de Zone d’Abomey Calavi/Sô-Ava” (CHU-ZAS) in Abomey-Calavi, located 25 km north of Cotonou. Children included in this study were aged between 2 and 6 years old and suffered from CM defined by deep coma (Blantyre score < 2) with *P. falciparum* infection and no other known cause of coma (e.g., acute bacterial meningitis, coma related to hypoglycaemia reversed by glucose infusion, status epilepticus, pre-existing neurological disease, traumatic or toxic coma). A negative HIV rapid diagnostic test and parental informed consent were also required for children to participate in the study.

### Clinical examination, blood sampling and child follow-up

Once consent was obtained, a thorough clinical examination was carried out, and venous blood samples were collected for research, haematologic and biochemistry analyses (ethylene diamine tetraacetie acid and heparin tubes), and blood culture (BD Bactec™ bottle, Becton Dickinson France). Blood samples were stored at 4 °C and then transferred within 4 h to the appropriate laboratory (medical or research laboratory). A lumbar puncture was performed as soon as possible if the patient was sufficiently clinically stable. Part of the sample was transferred to the medical analysis laboratory for culture, and part was stored at − 80 °C for subsequent pathogen testing. Patient urine was also collected during the day of inclusion (D0) and stored at − 80 °C as soon as possible. Urine was stored in 0.005% butylated hydroxytoluene to avoid oxidation. Finally, a fundoscopic examination (Eyepax 1.0 Dioptrix) was carried out on D1 to identify the retinopathies generally observed during CM and whose severity reflects the neurological sequelae [3]. The children were monitored clinically every day for the duration of their hospitalization. A thorough clinical examination was performed on the day of discharge from the hospital and 30 days after inclusion (D30). At D3 and D30, venous blood samples and urine were collected again, stored, and transferred as before.

### Confirmation of *P. falciparum* infection and absence of coinfection

At inclusion, *P. falciparum* infection was confirmed by thick and thin blood smear Giemsa staining and parasite quantification. Parasitemia was calculated for each patient based on their total leukocyte count. In cases of conflicting results between the rapid test and blood smear, *P. falciparum* infection was confirmed by PCR, as described below.

To rule out coinfections, blood culture, Gram staining, and bacterial culture of cerebrospinal fluid were performed at the university hospital reference laboratory (Centre National Hospitalier Universitaire, Cotonou). Cerebrospinal fluid was also tested for the presence of meningitis or encephalopathy by multiplex PCR targeting 14 pathogens (*Escherichia coli* K1, *Haemophilus influenzae*, *Listeria monocytogenes*, *Neisseria meningitidis*, *Streptococcus agalactiae*, *S. pneumoniae*, cytomegalovirus, enterovirus, herpes simplex virus 1 and 2, human herpes virus 6, human parechovirus, varicella-zone virus, *Cryptococcus neoformans/gattii*) using the Meningitis/Encephalitis FilmArray^®^ panel (Biomérieux, Craponne, France). In addition, dengue virus, Chikungunya virus, West Nile virus, *Plasmodium* spp., *Rickettsia* spp., *Leptospira* spp., *Salmonella* spp. (FTD Tropical Fever core PCR, Fast Track Diagnostics, Luxembourg) and *Plasmodium* species (*P. falciparum, P. malariae*, *P. ovale* and *P. vivax—*FTD Malaria differentiation, Fast Track Diagnostics, Luxembourg) and IgM measles (Measles IgM Capture EIA, Clin-Tech Limited, UK) were retrospectively performed on the blood samples.

### Urinary biomarkers

Urinary concentrations of 15-F2t-isoprostane, leukotriene B4 (LTB4), lipoxine A4 (LXA4), the metabolites of prostanglandine E2 metabolite (PGEM), reduced and oxidized glutathione (GSH and GSSG) and creatinine were measured using commercial enzyme immunoassays kits (Oxford Biomedical Research for 15-F2t-isoprostane, LTB4, LXA4 and PGEM, Cayman Chemical for GSH/GSSG, BioVision for creatinine) for CM patients at D0, D3 and D30, according to the manufacturer’s protocol. As recommended by the manufacturer, the samples were pre-treated with β-glucuronidase for 15-F2t-isoprostane dosage to separate isoprostane from glucuronic acid, with this complexed form representing more than 50% of the isoprostane present in the urine [27]. Similarly, prior to PGEM dosage, for reasons of PEG2 instability, standards and urine samples were subjected to derivatization to quantify a stable derivative. Finally, for the measurement of oxidized glutathione (GSSG), standards and samples were pretreated with 2-vinylpyridine to derivatize GSH. A second assay in the absence of pretreatment was carried out to determine the concentration of total GSH and the subsequent amount of reduced glutathione (GSH) equal to total GSH–GSSG. The plate reader TECAN Infinite F200 pro and dedicated software were used for plate readings. Values of the different urinary biomarkers were normalized to the urinary creatinine concentrations for each patient.

### Plasma biomarkers

Using Luminex technology, plasma levels of 17 markers of pro- and anti-inflammatory responses were simultaneously measured. The Human Premixed Multi-Analyte Kit (LXSAHM-17, R&D Systems, Lille, France) was used according to the manufacturer’s recommendation by the Anexplo platform (Genotoul, Toulouse, France). The plasma concentration of soluble EPCR was determined by ELISA according to the manufacturer’s recommendations (DuoSet^®^ R&D Systems).

### Statistical analysis

The main outcome of the study was malaria mortality among CM children, whereas plasmatic and urinary biomarkers were the first explanatory variables. The following variables were used for adjusting the different analyses: sociodemographic variables (age, sex), clinical variables (presence of hepatomegaly, splenomegaly, underweight, Blantyre score, history of vaccination, antibiotic use), and biological variables (blood smear, haemoglobin level, blood count, glycaemia, renal markers).

We described the general characteristics of children with CM and distinguished those who died or survived. For descriptive analysis, quantitative variables were presented as the means ± standard deviations or medians interquartile ranges, and qualitative variables were presented as frequencies (percentages). The variations in biomarkers between CM children who died and those who survived were assessed by using the nonparametric Mann‒Whitney U test for quantitative variables and the Pearson’s Chi2 test for qualitative variables.

We used the median for defining the thresholds to transform biomarkers into binary variables. Logistic regression models were performed to identify sociodemographic, clinical, biological and immunological biomarkers associated with malaria severity and mortality. Variables with a *P*-value < 0.20 in univariate analysis based on Fischer’s exact test were included in the multivariable analyses.

We conducted adjusted analyses separately for urinary and plasmatic biomarkers. For both biomarkers, multivariate analyses were performed in two steps. First, we identified demographic, clinical and biological variables that were significantly related to the study outcomes, and then we assessed urinary and plasmatic biomarkers that were also significantly associated with malaria mortality to take into account the interaction between biomarkers when appropriate. These statistically significant biomarkers from the multivariate analyses were adjusted for demographic, clinical and biological variables.

A manual backwards selection procedure was performed, and statistical significance was set at *P*-value < 0.05. All analyses were conducted using Stata 16.0 Software for Windows (Stata Corp, College Station, TX, USA).

## Results

### Sociodemographic and clinical characteristics of the study children

**Fig. 1:**
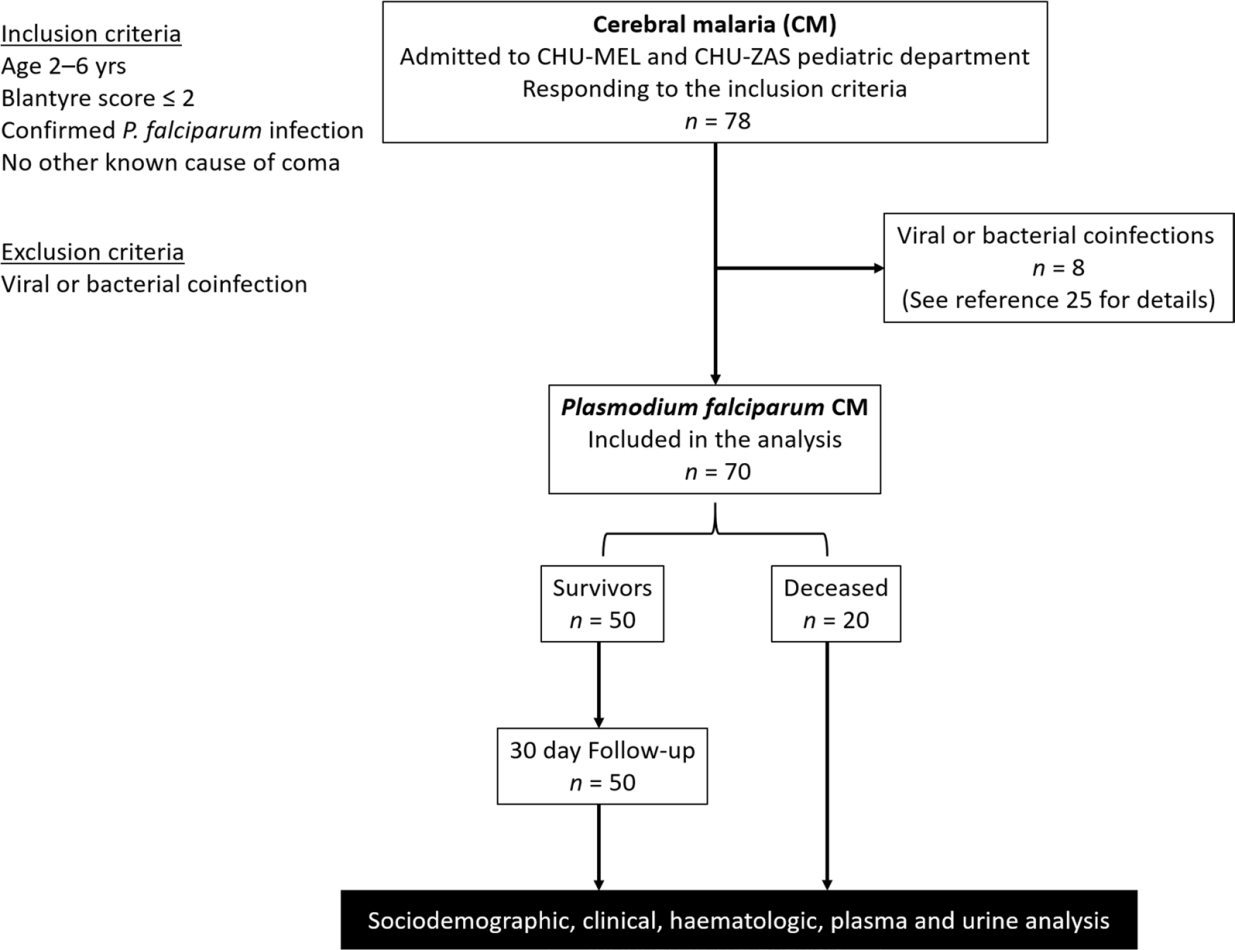
Flow diagram of the study participants. This study is part of a larger project called NeuroCM, studying non-traumatic coma in children. CM children were recruited from CHU-MEL and CHU-ZAC pediatric departments, two hospital from southern Benin. Here, the study focused on CM due to *Plasmodium falciparum* infection. Patients were excluded due to coinfections. Sociodemographic and clinical data were collected and analysed, as well as urine and blood samples for haematologic and plasma analysis at day 0 and day 30 for survivors

Initially, 78 children diagnosed with CM (deep coma and *P. falciparum* infection) were included in the NeuroCM study. Further analysis revealed 8 coinfections combining *falciparum* malaria with viral or bacterial infection, as previously described [25]. In this study, the 70 subjects with CM due to *P. falciparum* infection only were analysed and divided according to the clinical outcome into 50 children who survived and 20 who died (Fig. 1). The baseline characteristics of these patients were described and compared according to the group (Table 1). A majority of girls were enrolled (58.6% versus 41.4%), but sex had no incidence on disease outcome. The mean age was 45.04 (± 12.1) months. Children belonged mostly to the Fon ethnic group, the majority ethnic group in southern Benin. Among the sociodemographic and clinical factors studied here, five clinical features were associated with mortality, as shown in Table 1: no previous antibiotic treatment (*P* = 0.02), a lower Blantyre score (*P* = 0.03), symptoms of splenomegaly (*P* = 0.03 for both), jaundice (*P* = 0.004) and hyperlactatemia (*P* = 0.007).

**Table 1:** Sociodemographic characteristics of the NeuroCM study population, care pathway and clinical data at inclusion

Factors	Overall *n* = 70	Surviving children *n* = 50	Deceased children *n* = 20	*P*-value^a^
Female sex, *n*/*n* total (%)	41/70 (58.6)	31/50 (62.0)	10/20 (50.0)	0.36
Male sex, *n*/*n* total (%)	29/70 (41.4)	19/50 (38.0)	10/20 (50.0)	
Age in months, mean (± *SD*)	45.0 (± 12.1)	44.7 (± 12.1)	45.8 (± 12.3)	0.81
Ethnic group, *n*/*n* total (%)				
Adja	7/68 (10.3)	6/49 (12.2)	1/19 (5.3)	0.39
Aizo	8/68 (11.8)	8/49 (16.3)	0	0.06
Dendi	1/68 (1.5)	0	1/19 (5.3)	0.10
Fon	34/68 (50.0)	21/49 (42.9)	13/19 (68.4)	0.06
Goun	4/68 (5.9)	3/49 (6.1)	1/19 (5.3)	0.89
Weme	1/68 (1.5)	1/49 (2.0)	0	0.53
Xwla	2/68 (2.9)	1/49 (2.0)	1/19 (5.3)	0.48
Yoruba	2/68 (2.9)	2/49 (4.1)	0	0.37
Other	9/68 (13.2)	7/49 (14.3)	2/19 (10.5)	0.68
Undernet use, *n*/*n* total (%)	62/69 (89.9)	44/49 (89.8)	18/20 (90.0)	0.98
Antibiotics use, *n*/*n* total (%)	20/70 (28.6)	18/50 (36.0)	2/20 (10.0)	**0.02**
No measle vaccination, *n*/*n* total (%)	14/67 (20.9)	9/48 (18.7)	5/19 (26.3)	0.49
No yellow fever vaccination, *n*/*n* total (%)	18/67 (26.9)	11/48 (22.9)	7/19 (36.8)	0.25
Weight in kg, mean (± *SD*)	12.6 (± 2.4)	12.6 (± 2.5)	12.5 (± 2.4)	0.86
°C axillary temperature, mean (± *SD*)	38.3 (± 1.1)	38.4 (± 1.1)	38.0 (± 1.0)	0.21
Pulse /min, mean (± *SD*)	150.6 (± 21.0)	151.6 (± 20.6)	148.1 (± 22.6)	0.46
Blantyre score, mean (± *SD*)	1.7 (± 0.5)	1.8 (± 0.4)	1.4 (± 0.6)	**0.03**
Abnormal eyes fund, *n*/*n* total (%)	36/55 (65.5)	33/50 (66.0)	3/5 (60.0)	0.79
Hepatomegaly, *n*/*n* total (%)	37/70 (52.9)	26/50 (52.0)	11/20 (55.0)	0.82
Splenomegaly, *n*/*n* total (%)	25/70 (35.7)	14/50 (28.0)	11/20 (55.0)	**0.03**
Jaundice^b^, *n*/*n* total (%)	33/69 (47.9)	18/49 (36.7)	15/20 (75.0)	**0.004**
Hyperlactatemia^c^, *n*/*n* total (%)	42/70 (60.0)	25/50 (50.0)	17/20 (85.0)	**0.007**

^a^Mann‒Whitney U test for quantitative variables, Pearson’s *Chi2* test for qualitative variables

*P*-values in bold are < 0.05

^b^Clinical jaundice or total bilirubin > 29.2 mg/L(50 µmol/L)

^c^Hyperlactatemia means lactate > 5 mmol/L

### Parasitological, haematological and biochemical characteristics of the study population

The children enrolled were characterized by high parasitemia with a median parasitemia of 74,800/μl of blood and a low haemoglobin level with a mean of 58 (± 22) g/L (Table 2). Both variables had no effect on disease outcome. Among the biological factors studied, the number of leucocytes, and more particularly lymphocytes and monocytes, was higher in the group of deceased children (Table 2, *P* = 0.04, 0.01 and 0.004, respectively). For biochemistry factors, higher creatinine levels were associated with death (Table 2, *P* = 0.02).

**Table 2:** Parasitological, haematological and biochemistry characteristics of the NeuroCM study population

Factors	Overall *n* = 70	Surviving children *n* = 50	Deceased children *n* = 20	*P*-value^a^
*P. falciparum* density /µl of blood, median (IQR)	74,800 (12,425–421,278)	45,000 (4940–317,250)	296,000 (32,590–684,190)	0.09
Haemoglobin level in g/dL, mean (± *SD*)	5.8 (± 2.2)	5.9 (± 2.1)	5.7 (± 2.6)	0.71
Haemoglobin level < 11, % (*n*)	97.1 (68)	98.0 (49)	95.0 (19)	0.55
Hematocrit, mean (± *SD*)	17.1 (± 6.9)	17.4 (± 6.4)	16.6 (± 8.0)	0.42
Leucocytes × 10^3^/µl, mean (± *SD*)	16.8 (± 11.3)	15.9 (± 11.6)	18.7 (± 10.0)	**0.04**
Lymphocytes × 10^3^/µl, mean (± *SD*)	5.7 (± 4.5)	5.3 (± 4.7)	6.7 (± 3.5)	**0.01**
Monocytes × 10^3^/µl, mean (± *SD*)	0.9 (± 0.8)	0.9 (± 0.9)	1.2 (± 0.6)	**0.004**
Neutrophils × 10^3^/µl, mean (± *SD*)	9.9 (± 7.1)	9.6 (± 6.7)	10.5 (± 7.9)	0.62
Eosinophils × 10^3^/µl, mean (± *SD*)	0.2 (± 0.2)	0.2 (± 0.2)	0.2 (± 0.2)	0.64
Basophils × 10^3^/µl, mean (± *SD*)	0.001 (± 0.01)	0.002 (± 0.01)	0	0.90
Thrombocytes × 10^3^/µl, mean (± *SD*)	111.5 (± 98.3)	105.04 (± 82.7)	131.3 (± 137.5)	0.99
Glucose in g/L, mean (± *SD*)	0.8 (± 0.7)	0.9 (± 0.7)	0.5 (± 0.5)	0.07
Glucose < 0.4 g/L, % (*n*)	35.7 (25)	22.0 (11)	70.0 (14)	0.17
Urea in g/L, mean (± *SD*)	0.2 (± 0.2)	0.2 (± 0.1)	0.3 (± 0.3)	0.09
Creatinine in mg/L, mean (± *SD*)	6.2 (± 7.2)	4.8 (± 3.5)	10.2 (± 12.3)	**0.02**

*SD*: Standard deviation; *IQR* Interquartile range

^a^Mann‒Whitney U test for quantitative variables, Pearson’s *Chi2* test for qualitative variables

*P*-values in bold are < 0.05

### Higher IL-8 levels are strongly associated with cerebral malaria death

**Fig. 2:**
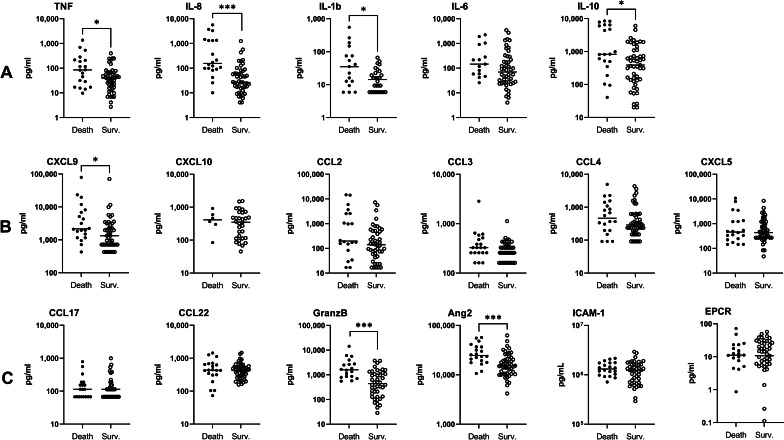
Plasma biomarker expression levels in children who survived and died. Levels of cytokines, chemokines and markers of endothelial damage and activation were measured in the plasma of children at inclusion (D0) by the Luminex assay and compared between surviving (*n* = 50) and deceased children (*n* = 20) by the Mann‒Whitney U test. Values are all in pg/ml. **A** TNF, IL-8, IL-1β, IL-6 and IL-10 levels. **B** CXCL9, CXCL10, CCL2, CCL3, CCL4, CXCL5. **C** CCL17, CCL22, granzyme B, angiopoietin-2, ICAM-1 and EPCR levels. *P < 0.05, **P < 0.005, ***P < 0.0005. Surv.: Survival

**Fig. 3:**
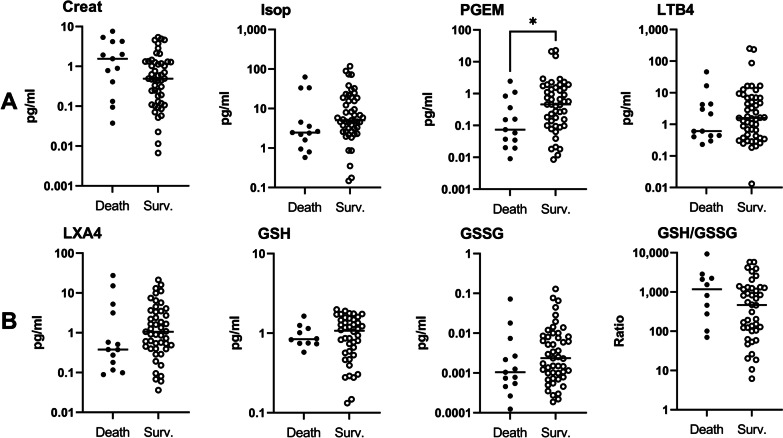
Urinary biomarker expression levels in children who survived and died. Levels of creatinine, 15-F2t-isoprostane (Isop), PGE2 metabolites (PGEM), LTB4, LXA4, reduced (GSH) and oxidized (GSSG) glutathione were measured in the urine of children at inclusion (D0) by enzyme immunoassays kits and compared between surviving (*n* = 50) and deceased children (*n* = 20) by the Mann‒Whitney U test. Values are all in pg/ml. **A** Creatinine, 15-F2t-isoprostane, PGEM, and LTB4 levels. **B** LXA4, GSH, GSSG and GSH/GSSG ratio levels. *P < 0.05, **P < 0.005, ***P < 0.0005. Surv.: Survival

To identify specific risk factors for CM mortality among the mediators of the cellular immune response, we then analysed the relationship between clinical outcome and 24 immune parameters, including 17 plasma and 7 urinary factors. Figures 2 and 3 present the univariate analysis using Mann‒Whitney U tests for plasma and urinary parameters. The results showed that plasma levels of the proinflammatory cytokines TNF, IL-8, and IL-1β and the anti-inflammatory cytokine IL-10 were higher in children who died, compared to the levels observed in the children who survived, with *P*-values of Mann‒Whitney *U* tests between 0.03 and < 0.0001 (Fig. 2A). Among chemokines, higher plasma CXCL9 levels, implicated in Th1, CD8 LT and NK trafficking, were associated with death outcome (Fig. 2B, *P* = 0.02). Higher plasma levels of granzyme B and angiopoietin-2, a marker of endothelium activation, were also associated with death outcome (Fig. 2C, *P* = 0.002 and 0.0005, respectively). Interestingly, for urinary markers, increased levels of PGEM, a marker of PGE2 biosynthesis and an inhibitor of the inflammatory response, were related to survival (Fig. 3A, *P* = 0.04). To consolidate our results, we then performed logistic regression to identify the risk factors for death among the plasma and urinary parameters measured in two distinct models (Tables 3 and 4). Table 3 demonstrates that levels equal to or above 57.5 pg/ml of plasma IL-8 constituted a high risk of death during CM (*aOR* = 14.2, *P* = 0.002). The regression analysis did not reveal any other risk factor for death among the plasma factors tested. For the urinary parameters, PGEM levels equal to or above 0.43 ng/mg creatinine constituted a protective factor against CM death with an adjusted odds ratio of 0.31, but this result was not significant (Table 4, *P* = 0.14).

**Table 3:** Immunological factors measured in plasma associated with death among cerebral malaria infants, multivariate logistic regression

Factors		Survival, % (*n*)	Death, % (*n*)	Univariate analysis		Multivariate analysis	
				*OR* (95% *CI*)	*P*-value	*aOR* (95% *CI*)	*P*-value
TNF (pg/ml)	< 41.4	79.4 (27)	20.6 (7)	1			
	≥ 41.4	62.9 (22)	37.1 (13)	2.27 (0.78–6.69)	0.13		
IL-8 (pg/ml)	< 57.5	91.7 (33)	8.3 (3)	1		1	
	≥ 57.5	48.5 (16)	51.5 (17)	11.7 (2.98–45.8)	**0.004**	14.2 (2.56–78.4)	**0.002**
IL-10 (pg/ml)	< 541.2	80.0 (28)	20.0 (7)	1			
	≥ 541.2	61.8 (21)	38.2 (13)	2.48 (0.84–7.28)	0.10		
CCL2 (pg/ml)	< 172.9	78.8 (26)	21.2 (7)	1			
	≥ 172.9	60.6 (20)	39.4 (13)	2.41 (0.81–7.17)	0.11		
Angiopoietin-2 (pg/ml)	< 17,774	88.6 (31)	11.4 (4)	1			
	≥ 17,774	52.9 (18)	47.1 (16)	6.89 (1.99–23.8)	**0.002**		
CCL4 (pg/ml)	< 336.6	78.1 (25)	21.9 (7)	1			
	≥ 336.6	61.8 (21)	38.2 (13)	2.21 (0.75–6.55)	0.15		
CXCL9 (pg/ml)	< 1823.1	80.6 (25)	19.4 (6)	1			
	≥ 1823.1	56.2 (18)	43.7 (14)	3.24 (1.04–10.1)	**0.04**		
Granzyme B (pg/ml)	< 792.4	87.5 (28)	12.5 (4)	1			
	≥ 792.4	53.3 (16)	46.7 (14)	6.12 (1.72–21.8)	**0.005**		

*OR* Odd ratio; *aOR* Adjsuted odd ratio, adjusted for Blantyre score, presence of icterus, hypoglycaemia, and blood creatinine; *CI* Confidence interval

*P*-values in bold are < 0.05

**Table 4:** Immunological factors measured in urine associated with death among cerebral malaria infant, multivariate logistic regression, *n* = 70

Factors		Survival, % (*n*)	Death, % (*n*)	Univariate analysis		Multivariate analysis	
				*OR* (95% *CI*)	*P*-value	*aOR* (95% *CI*)	*P*-value
Urinary creatinine (mg/ml)	< 0.60	87.1 (27)	12.9 (4)	1			
	≥ 0.60	70.9 (22)	29.0 (9)	2.76 (0.75–10.2)	0.13		
LXA4/creatinine (ng/mg)	< 0.79	68.9 (20)	32.3 (10)	1			
	≥ 0.79	87.1 (27)	12.9 (4)	0.31 (0.08–1.13)	0.08		
IsoP/creatinine (ng/mg)	< 4.63	68.7 (22)	31.3 (10)	1			
	≥ 4.63	90.0 (27)	10.0 (3)	0.24 (0.06–0.99)	0.05		
PGEM/creatinine (ng/mg)	< 0.43	67.7 (21)	32.3 (10)	1		1	
	≥ 0.43	89.7 (26)	10.3 (3)	0.24 (0.06–0.99)	0.05	0.31 (0.06–1.49)	0.14

*OR* Odd ratio; *aOR* Adjsuted odd ratio; adjusted for Blantyre score, presence of icterus, hypoglycaemia, and blood creatinine; *CI* Confidence interval

### Increasing levels of plasma CXCL5, CCL17 and CCL22 and urinary 15-F2t-isoprostane are associated with resolution of CM during follow-up

**Fig. 4:**
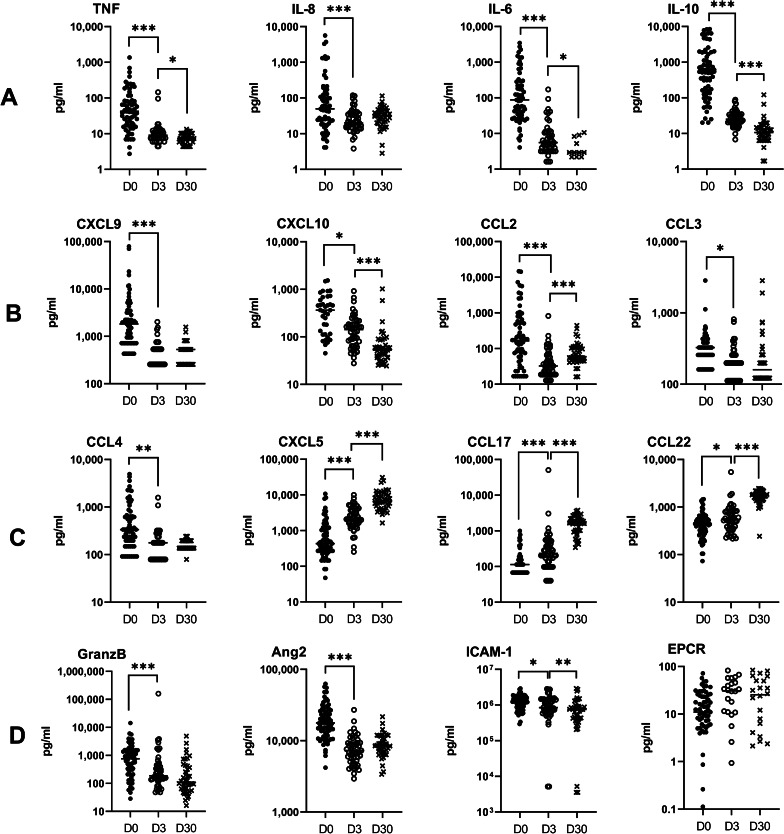
Kinetics of plasma biomarkers during and after CM (surviving children at D0, D3, D30). Levels of cytokines, chemokines and markers of endothelial damage and activation were measured in the plasma of children at inclusion (D0) and 3 (D3) and 30 days (D30) after inclusion by Luminex assay. Two-by-two comparisons (D0 to D3 and D3 to D30) were carried out by the Wilcoxon matched-pairs signed rank test. Values are all in pg/ml. **A** TNF, IL-8, IL-6 and IL-10 levels. **B** CXCL9, CXCL10, CCL2, CCL3. **C** CCL4, CXCL5, CCL17, CCL22. **D** Granzyme B, angiopoietin-2, ICAM-1 and EPCR levels. ^*^P < 0.05, ^**^P < 0.005, ^***^P < 0.0005

**Fig. 5:**
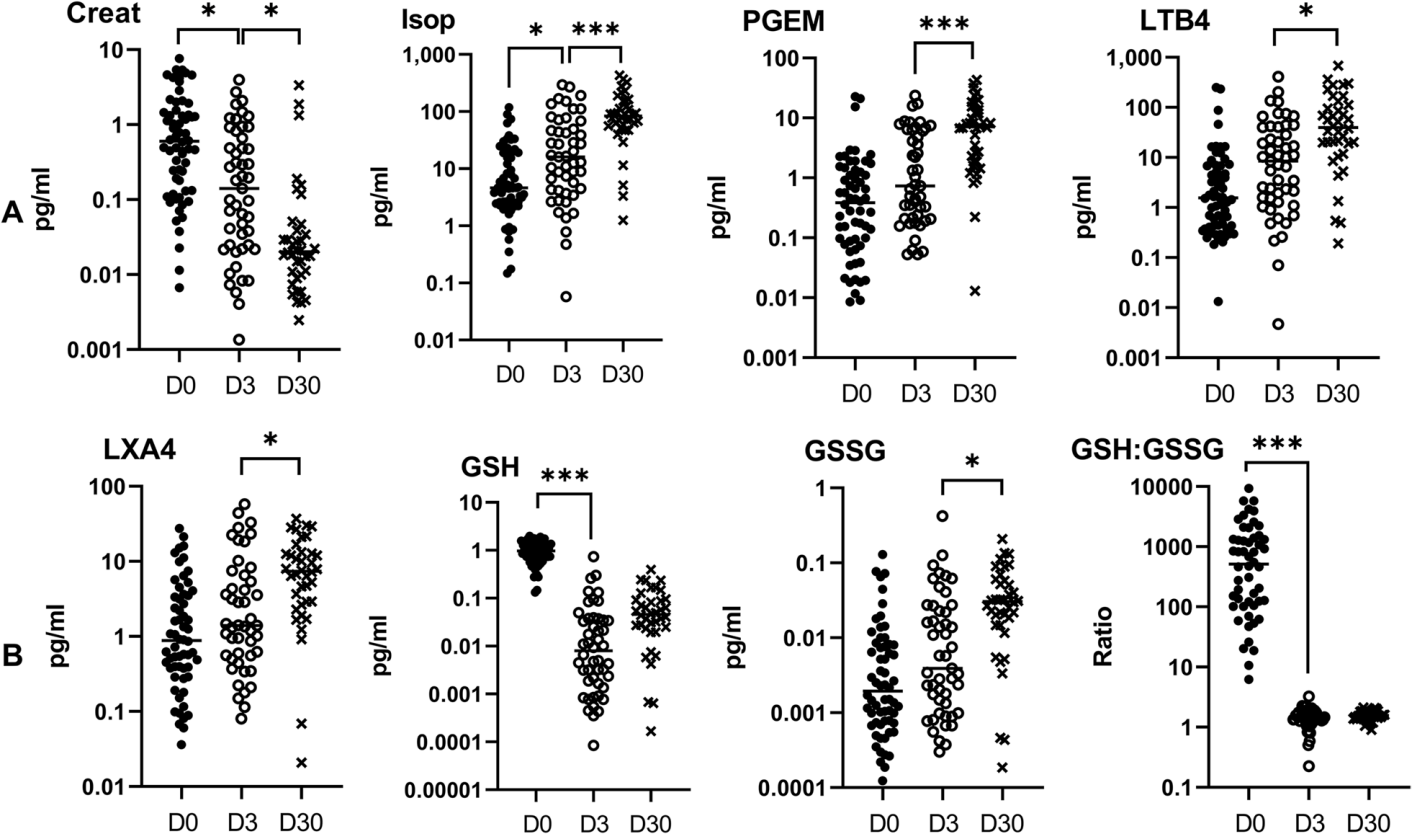
Kinetics of urinary biomarkers during and after CM (surviving children at D0, D3, D30). Levels of creatinine, 15-F2t-isoprostane (Isop), PGE2 metabolites (PGEM), LTB4, LXA4, reduced (GSH) and oxidized (GSSG) glutathione were measured in the urine of children at inclusion (D0) and 3 (D3) and 30 days (D30) after inclusion by enzyme immunoassays kits. Two-by-two comparisons (D0 to D3 and D3 to D30) were carried out by the Wilcoxon matched-pairs signed rank test. Values are all in pg/ml. **A** Creatinine, 15-F2t-isoprostane, PGEM, and LTB4 levels. **B** LXA4, GSH, GSSG and GSH/GSSG ratio levels. *P < 0.05, ^**^P < 0.005, ***P < 0.0005

Plasma and urinary values were then followed at D3 and D30 after inclusion (Figs. 4, 5). Plasma levels of TNF, IL-6, IL-10, CXCL10 and ICAM-1 gradually decreased at the three follow-up points (Fig. 4A, B, D), similar to urinary creatinine (Fig. 5A). Conversely, plasma levels of CXCL5, CCL17, and CCL22, as well as urinary 15-F2t-isoprostane, increased from D0 to D30 (Figs. 4C, 5A). Interestingly, plasma IL-8, which was found to be a risk factor for death via regression analysis, showed rapidly decreasing levels, as the values displayed at D3 and D30 were similar [median (25th–75th percentile), 20.1 (14.9–37.0) and 31.3 (19.9–44.4), respectively] but much lower than those at D0 [49.6 (18.3–148)] (Fig. 4A). This phenomenon was also observed for plasma CXCL9, CCL3, CCL4, granzyme B, angiopoietin-2, urinary GSH and the urinary GSH∶GSSG ratio (Figs. 4B–D and 5B). The evolution of this ratio reflects a more pronounced antioxidant state at D0 than at D3 and D30, as well as the evolution of 15-F2t-isoprostane levels during follow-up (Fig. 5A). Urinary levels of PGEM, LTB4, LXA4 and GSSG were similar at D0 and D3 and displayed an increase at D30 (Fig. 5A and B), which reflects either a longer impact of the disease on their levels or a specific involvement in the resolution phase. Finally, CCL2 showed a fluctuating level during monitoring, with a level that dropped sharply from D0 to D3 and then increased from D3 to D30 (Fig. 4B). However, the level measured at D30 remained much lower than that observed at D0 (*P* < 0.0001).

## Discussion

The aim of this study was to identify predictive factors of death or survival during CM. To this end, 70 Beninese children with CM (50 who survived and 20 who died) were included in the present study, which is part of a larger project named NeuroCM [26]. In this analysis, we chose to focus only on subjects with CM. This choice of analysis, which excluded UM subjects, allowed us to work on a clinically homogeneous group and to identify relevant risk factors for death.

An accurate differential diagnosis was achieved through thorough clinical examination, including fundoscopic examination and extensive blood biochemical analysis associated with the molecular diagnosis of microbial infections in the patients’ cerebrospinal fluid. Fundoscopic examination was difficult to perform and could not be performed on the children with the most severe conditions; the data are rather indicative. This approach resulted in a homogeneous group of CM patients thanks to the exclusion of coinfections and nonmalarial comas. In a previous paper dealing with the NeuroCM study, a multivariate analysis revealed that high bilirubin (jaundice) and lactate levels were predictive of early death in Beninese children with nontraumatic coma, whereas the use of antibiotics before admission and yellow fever vaccination were protective factors [25]. In our study, similar associations were found in the univariate analysis, except for vaccination against yellow fever. The similar results obtained for jaundice, hyperlactatemia and previous antibiotic use suggest that such a clinical profile is more specific to coma, including CM-related coma, than to CM itself. Interestingly, a lower Blantyre score and splenomegaly were also identified as risk factors for CM death. A more pronounced coma was previously described as a risk factor for death during severe malaria in children [28], in adults presenting with CM [29], and in children presenting with CM [30, 31]. Splenomegaly is also a clinical manifestation classically described in cerebral malaria [32, 33], although rarely identified as a risk factor for death during CM. Through the analysis of haematologic factors, we observed higher numbers of leukocytes, among which lymphocytes and monocytes were associated with CM death, indicating an increased immune response related to the infection. Higher creatinine levels were also associated with death in CM children, suggesting that renal damage was involved in the death of patients.

We then analysed death risk factors among specific plasma and urinary mediators of both inflammatory and oxidative responses. We found by univariate analysis that plasma levels of TNF, IL-8, IL-1β, IL-10, CXCL9, granzyme B and angiopoietin-2 measured at the acute phase (D0) were higher in children who died compared to those who survived. For urinary mediators, lower levels of PGEM at D0 were associated with CM death through univariate analysis. However, a higher initial level of plasma IL-8 alone was identified as a risk factor for death by multivariate analysis. Plasma and urinary levels measured during convalescence (D3 and D30) confirmed these trends and suggested two kinds of marker profiles: a disease severity profile for decreasing markers including plasma TNF, IL-8, IL-10, CXCL9, granzyme B, angiopoietin-2, along with plasma IL-6, CXCL10, CCL2, CCL3, CCL4, ICAM-1, and urinary creatinine, GSH and GSH∶GSSG ratio; and a disease resolution profile for increasing markers including plasma CXCL5, CCL17, CCL22, and urinary PGEM, 15-F2t-isoprostane, LTB4, LXA4, and GSSG. It is important to note that the values obtained at D3 and D30 all belong to surviving children, as all deaths occurred before D3.

In line with our results, previous studies have reported associations between malaria severity or death and proinflammatory cytokines and the anti-inflammatory cytokine IL-10 [34–40]. During CM, proinflammatory cytokines such as TNF and IL-1β are thought to induce endothelial activation, as demonstrated in vitro [41, 42], and to play a major role in iRBC sequestration in cerebral micro vessels [14, 43] through overexpression of adhesion molecules (ICAM-1, VCAM), accumulation of cytotoxic CD8 + T cells and alteration of the BBB [14, 44]. An increase in plasma IL-1β along with IL-10 and TNF has also previously been associated with CM outcome [37]. However, the implication of IL-10, an anti-inflammatory cytokine involved in the inhibition of proinflammatory responses and the development of regulatory responses, is discussed more. It has been shown in mice that the absence of IL-10 expression was associated with the development of severe malaria and excess mortality, suggesting a protective role [45, 46], whereas high plasma IL-10 levels in humans were correlated with malaria severity and death [14, 39, 47]. Our results are in line with most human studies suggesting a role for high plasma IL-10 in malaria severity and CM death.

Among the plasma markers measured, high levels of IL-8 constituted the most significant risk of death (odds ratio 14.2) in our study. Other studies also reported a significant association of high levels of plasma or serum IL-8 with severity or death among severe cases [severe malarial anaemia (SMA) or cerebral malaria] [40, 48]. Concerning more specifically CM, Pappa et al. showed a positive and significant correlation between plasma IL-8 levels and brain volume in CM children [49]. Such a correlation was also found for TNF, CCL2 and IL-10, in line with our results. Interestingly, elevated cerebrospinal IL-8 levels, but not serum IL-8 levels, were identified as a marker of CM death among children who died of CM, SMA, or nonmalaria causes [50]. However, in this study, samples were collected after death. Finally, a recent study reported higher levels of IL-8 in retinopathy-positive CM children who died than in survivors and UM children, similar to our results [51]. The authors claim that retinopathy diagnosis improves clinical CM specificity, as it is related to iRBC sequestration in brain. In our case, we think that our diagnosis strategy was sufficiently thorough to ensure the selection of real CM cases. IL-8 is a chemokine whose main function is the promotion of neutrophil activation and migration to sites of inflammation; activated neutrophils also produce IL-8 themselves, reinforcing the inflammatory loop [13]. During CM, neutrophils can play both a beneficial role by killing blood-stage parasites [52] but also a deleterious role through ROS production and toxic mediator release such as neutrophil extracellular traps (NETs), leading to an alteration of the brain endothelium and increased iRBC sequestration [53–56]. Plasma levels of NET components were shown to be higher in severe malaria patients and to promote parasite sequestration and tissue damage in a murine malaria model [56]. Although ECM studies report leucocyte accumulation in the brain [57], autopsies carried out in humans after CM death rarely reported neutrophil accumulation in brains [58–60], suggesting a putative effect of neutrophils through their activation and ability to produce soluble factors rather than their migration to inflammation sites. Thus, high IL-8 levels may mostly participate in neutrophil activation. In addition, a disease resolution profile was obtained for CXCL5, a chemokine implicated in neutrophil trafficking [13], with increasing levels from D0 to D30 in surviving children. This results reinforce our hypothesis of the importance of neutrophil activation rather than migration to inflammation sites. Interestingly, IL-8 has also been identified as a pharmacological target to reduce ischaemia-induced myocardial injury [61], suggesting that decreasing or neutralizing plasma IL-8 could help reduce neuroinflammation and ischaemia during CM.

In our study, among chemokines other than IL-8, only the level of plasma CXCL9 was related to CM death. This is a surprising result, as high levels of CXCL10 and CCL2 have been frequently reported as markers of severity and death during malaria and even CM [36, 39, 62, 63]. For CXCL10, the low number of doses carried out successfully may explain our result. To our knowledge, this is the first time that a study highlights a correlation between CXCL9 and death in CM patients. However, its deleterious implication during ECM has already been demonstrated [64], and an increase in its gene expression has been observed in the brains of infected mice [65]. CXCL9 is known for its involvement in CD8 T-cell migration to the brain and in their activation leading to the release of granzyme B and brain endothelium alteration, as demonstrated in ECM [66, 67]. Two recent studies have highlighted the involvement of CD8 T cells in human CM through their localization in the brains of Malawian children who died of CM [68, 69]. Kaminski et al. also observed higher plasma levels of granzyme B during malaria infection compared to healthy patients and an increased proportion of blood CD8^+^/GrzB^+^ T cells in severe malaria patients [70]. Such results combined with ours support the idea that CXCL9 and granzyme B are players in the neuroinflammatory response during CM. Conversely, CCL17 and CCL2 which are known for their implication in Th2 response and regulatory T cell migration [13], were found to be associated with disease resolution in surviving children.

Elevated plasma angiopoietin-2 and soluble ICAM-1 are two markers of endothelium activation and increased permeability that were previously associated with malaria severity and risk of cognitive impairment [71, 72]. In our study, elevated levels of angiopoietin-2, a marker of increased vascular permeability, were also found in patients who died compared to those who survived. However, the levels of plasma soluble ICAM-1 did not differ between groups, suggesting that the level of angiopoietin-2 is a better prognostic marker of death than the level of soluble ICAM-1.

In urine, we measured some metabolites of arachidonic acid known for their implication in the inflammatory response. Among the metabolites measured, only PGEM was related to CM death with a disease resolution profile, i.e., with lower initial urinary levels in deceased subjects than in surviving subjects. PGEM is the major metabolite of PGE2, which is unstable in vivo and produced via the activation of the enzyme COX-2 [73]. Other teams previously reported lower levels of urinary bicyclo-PGE2, another PGE2 metabolite, in severe malarial anaemia patients and CM patients than in those presenting uncomplicated or asymptomatic malaria [23, 24]. These authors also demonstrated that reduced PGE2 levels were related to downregulation of COX-2 and enhanced uptake of haemozoin by monocytes. In our study, the large number of patients (70 CM subjects in total, of which 20 died and 50 survived) probably helped in identifying a significant relationship between the occurrence of death during CM and low levels of urinary PGEM. In addition, it is important to emphasize that the urinary determination of biological parameters in CM children is an interesting approach because of its ease of implementation and its non-invasive character.

Finally, the evolution of 15-F2t-isoprostane levels and the GSH∶GSSG ratio in urine during follow-up of CM children in our study reflects a more pronounced antioxidant balance at D0 and a re-establishment of a more prooxidant response over the course of the follow-up among CM children who survived. However, these lower levels observed at inclusion were not related to death outcome. This result is surprising, as oxidative damage is thought to contribute to malaria pathogenicity [74]. Additionally, higher levels of urinary F2-isoprostane metabolites were previously reported in patients presenting severe malaria and acute kidney injury than in patients with non-severe malaria [75, 76]. However, our study is the first to analyse the evolution of oxidative markers in urine among CM children. A defect in the oxidative response by the leukocytes of these children could explain these results and provide a new avenue for research.

Our study presents two main limitations: the relatively small number of 70 subjects with CM, which allowed analyses comparing 50 subjects who survived to 20 who died; and the point of biological follow-up at D3 post-inclusion. A posteriori, D3 proved to be too late to measure the evolution of markers in the group of subjects who did not survive, as deaths occurred within the first two days after inclusion in the study. Therefore, marker changes at D3 and D30 only relate to the group of subjects who survived CM.

## Conclusions

Multivariate logistic regression highlighted elevated plasma levels of IL-8 as the main risk factor for death during CM. Follow-up of patients at D3 and D30 revealed marker profiles of disease aggravation and resolution for markers implicated in neutrophil activation, endothelium activation and damage, inflammatory and oxidative response, including the striking increase of oxidative markers 15-F2t-isoprostane and GSH∶GSSG ratio. These results provide important insight into our understanding of CM pathogenesis and clinical outcomes and may have important therapeutic implications.

## Data Availability

The datasets used during the current study are available with principal investigator.

## Abbreviations

*aOR*: Adjusted odds ratio
BBB: Blood brain barrier
CCL: C–C motif chemokine ligand
CHU-MEL: Centre Hospitalier Universitaire de la Mère et de l’Enfant Lagune
CHU-ZAS: Centre Hospitalier Universitaire de Zone d’Abomey Calavi/Sô-Ava
*CI*: Confidence interval
CM: Cerebral malaria
Creat: Creatinine
CXCL: C-X-C motif chemokine ligand
D0, D3, D30: Day 0, Day 3, Day 30
ECM: Experimental CM
EPCR: Endothelial protein C receptor
ICAM-1: Intercellular adhesion molecule-1
iRBCs: Infected red blood cells
IL: Interleukine
iRBC: Infected red blood cell
IQR: Interquartile range
*OR*: Odds ratio
Isop: 15-F2t-isoprostane
PCR: Polymerase chain reaction
PGE2: Prostanglandine E2
PGEM: Prostanglandine E2 metabolite
ROS: Reactive oxygen species
*SD*: Standard deviation
TNF: Tumour necrosis factor
UM: Uncomplicated malaria
